# Supplementary material for: Dynamical criticality of spin-shear coupling in van der Waals antiferromagnets
Source: Nat Commun. 2022 Nov 3;13:6598. doi: 10.1038/s41467-022-34376-5 (PMC9633802; doi:10.1038/s41467-022-34376-5)
Supplement: Supplementary file 1 — Supplementary Information [file 41467_2022_34376_MOESM1_ESM.pdf]

Supplementary Information for

Dynamical criticality of spin-shear coupling in van der Waals antiferromagnets

Faran Zhou[1], Kyle Hwangbo[2], Qi Zhang[1,2,9], Chong Wang[3], Lingnan Shen[2], Jiawei Zhang[1], Qianni Jiang[2], Alfred Zong[4], Yifan Su[5], Marc Zajac[1], Youngjun Ahn[1,6], Donald A. Walko[1], Richard D. Schaller[7], Jiun-Haw Chu[2], Nuh Gedik[5], Xiaodong Xu[2,3], Di Xiao[3,2], Haidan Wen[1,8*]

[1]X-ray Science Division, Argonne National Laboratory, Lemont, IL, USA

[2]Department of Physics, University of Washington, Seattle, WA, USA

[3]Department of Materials Science and Engineering, University of Washington, Seattle, WA, USA

[4]Department of Chemistry, University of California Berkeley, Berkeley, CA, USA

[5]Department of Physics, Massachusetts Institute of Technology, Cambridge, MA, USA

[6]Department of Materials Science and Engineering, University of Wisconsin-Madison, Madison, WI, USA

[7]Center for Nanoscale Materials, Argonne National Laboratory, Lemont, IL, USA

[8]Materials Science Division, Argonne National Laboratory, Lemont, IL, USA

[9]Present address: Department of Physics, Nanjing University, Nanjing, China.

*Correspondence to: wen@anl.gov

Table of contents

Supplementary Figures 1-9

Supplementary Notes 1-6

Supplementary Table 1

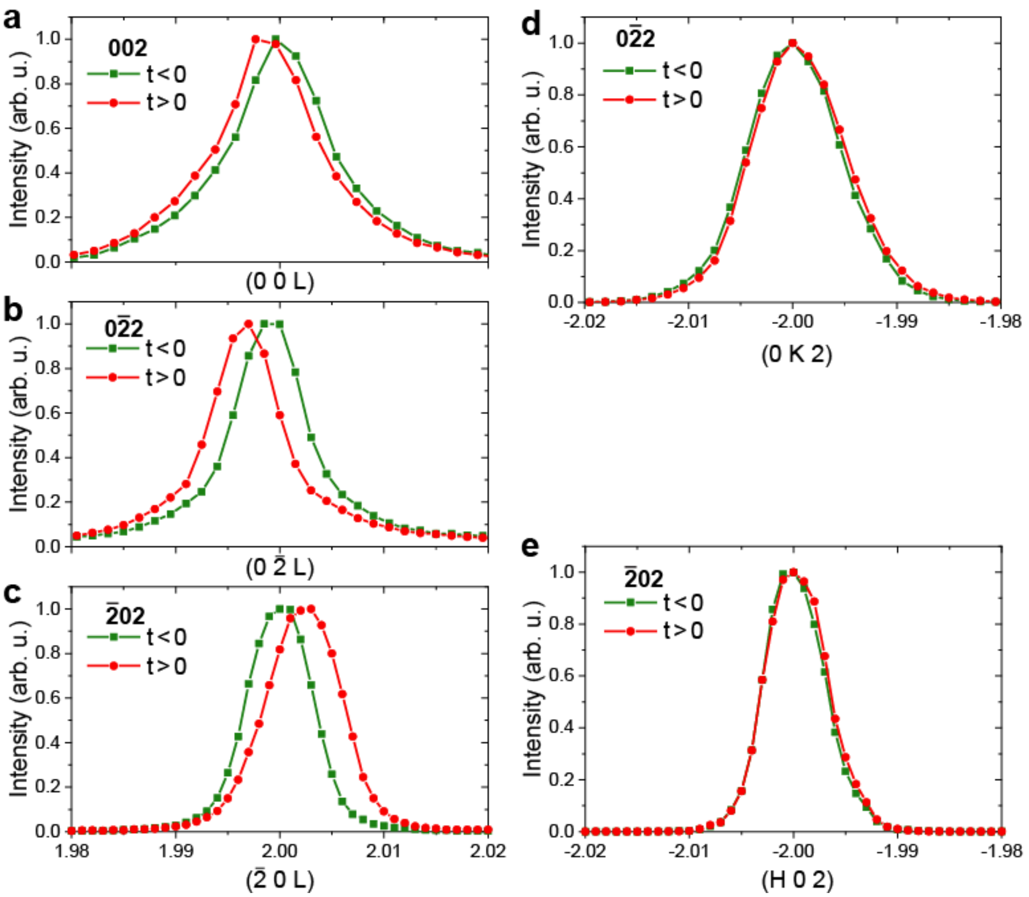

Supplementary Figure 1: HKL scans of 002, 0$\bar{2}$2, $\bar{2}$02 Bragg peaks of $FePS_3$ before and after laser excitation. HKL scans of 002 (**a**), 0$\bar{2}$2 (**b**, **d**), and $\bar{2}$02 (**c, e**) peaks before (t = -5 ns, green) and after (t = 25 ns, red) laser excitation at 95 K. The HKL scans were performed using SPEC program from Certified Scientific Software on a 6-circle diffractometer.

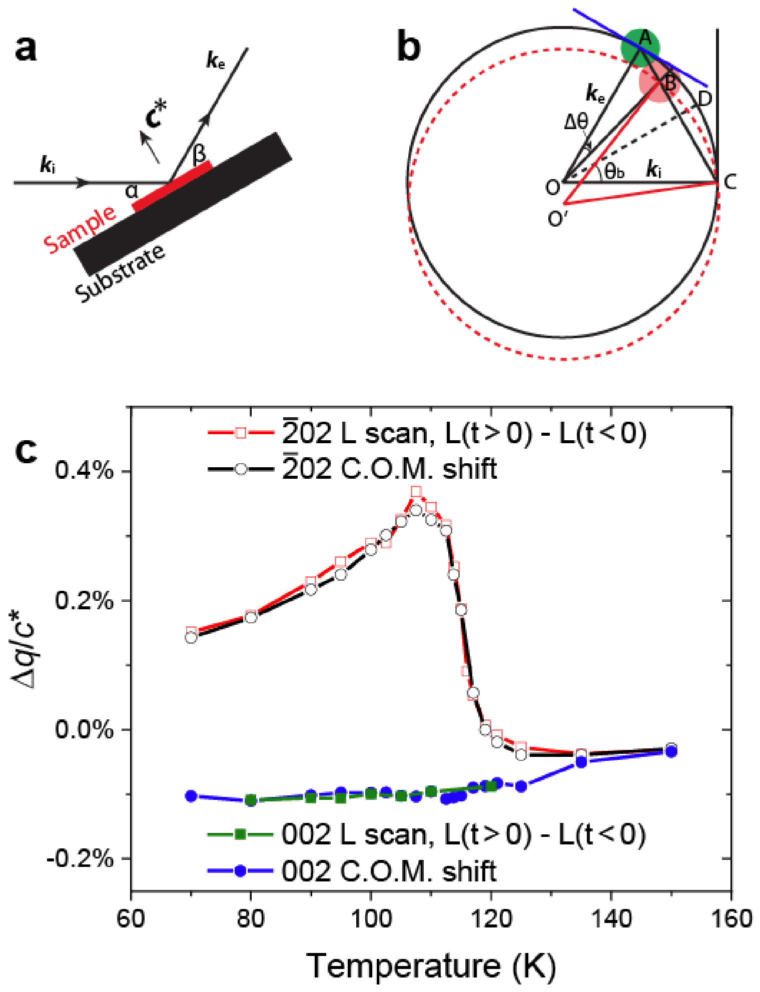

Supplementary Figure 2: Calibrating the peak shift measured by the center of mass on the detector in reciprocal unit. **a** Schematic of XRD reflection geometry. **b**, Schematic of the Ewald sphere and Bragg peaks in reciprocal space. Green and red disk: finite size Bragg peaks before and after laser excitation. Red dashed circle and red lines: Bragg condition for $t > 0$. **c**, The Bragg peak shifts as measured by the center of mass for 002 and $\bar{2}02$ peaks in $FePS_3$ are compared with the results obtained by the L scans. An example of L scan at 95 K is shown in Supplementary Figure 1.

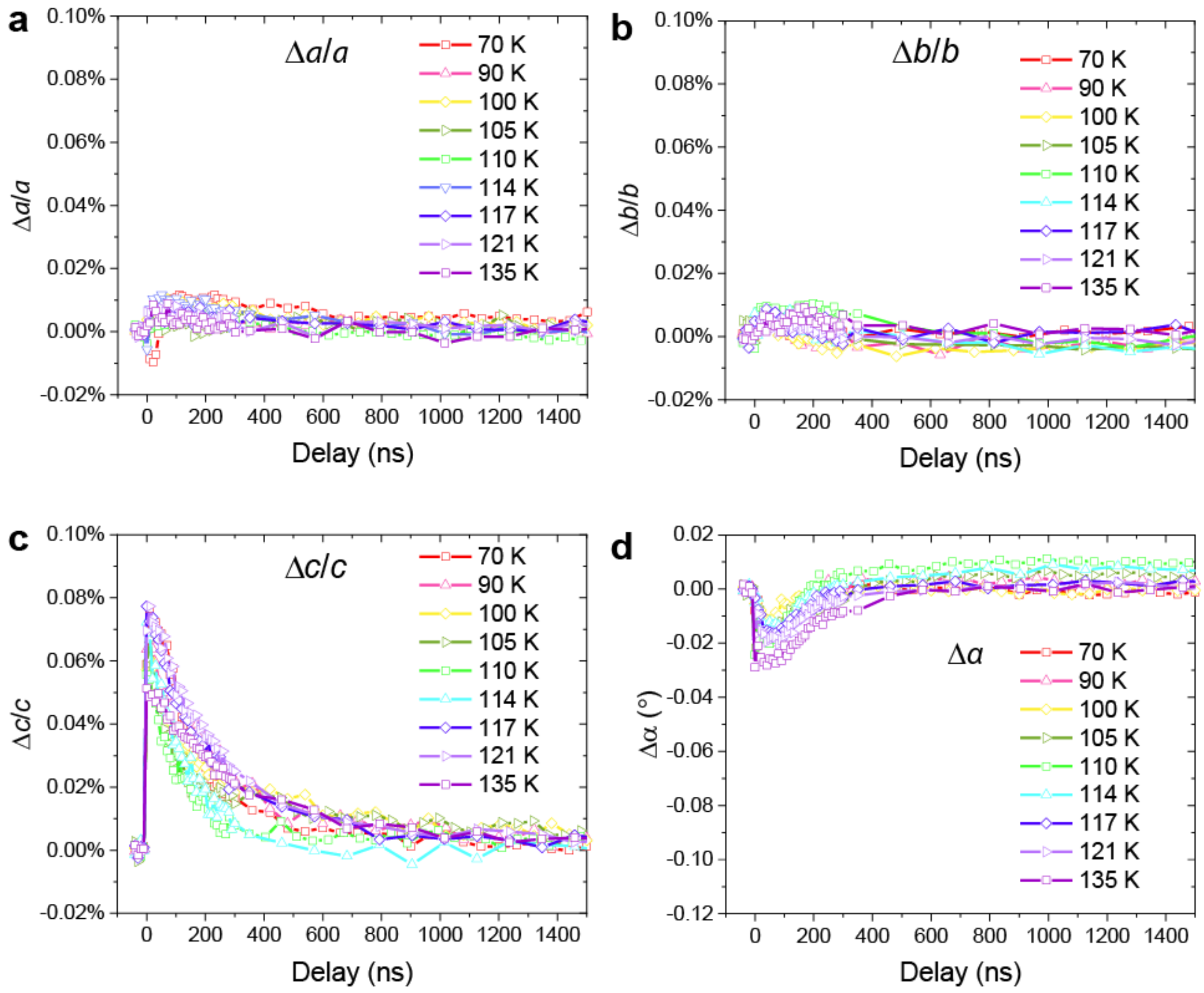

Supplementary Figure 3: Dynamics of $FePS_3$ lattice parameters a (**a**), b (**b**), c (**c**), and $\Delta\alpha$ (**d**). These parameters were calculated based on the shifts of $\bar{2}02$, $0\bar{2}2$, and 002 Bragg peaks. Monoclinic angle β was extracted from $\bar{2}02$ and 002 dynamics, detailed in Supplementary Note 3. Similarly, angle α was extracted from $0\bar{2}2$ and 002 dynamics. Lattice parameters were extracted based on: $a = \frac{2\pi}{\sin(\beta)\, a^*}$, $b = \frac{2\pi}{b^*}$, $c = \frac{2\pi}{\sin(\beta)\, c^*}$, $\Delta\alpha = \alpha(t) - \alpha(t < 0)$.

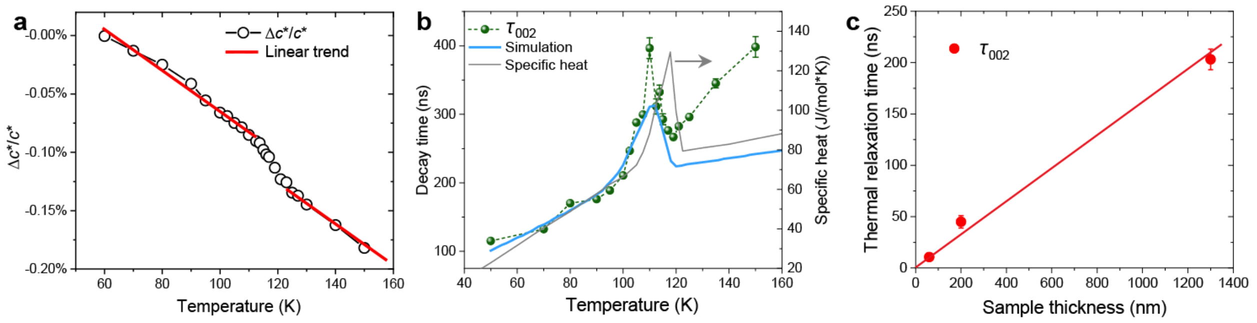

Supplementary Figure 4: Thermal expansion and relaxation of $FePS_3$. **a**, The measured percentage change of *c** as a function of temperature. The red lines represent the linear thermal expansion at temperatures below and above T_N. **b**, Simulation of the thermal relaxation time. The gray line is the specific heat adapted from Ref.[1]. The green data points are the relaxation time measured by c* change of 002 peak. The blue curve is the simulation results based on a 1D thermal transport model as described in Supplementary Note 2. The error bars represent the standard deviation of exponential fitting. **c**, Thickness-dependent thermal relaxation time measured at 100 K. The red line is a linear fit of the data. The error bars represent the standard deviation of exponential fitting.

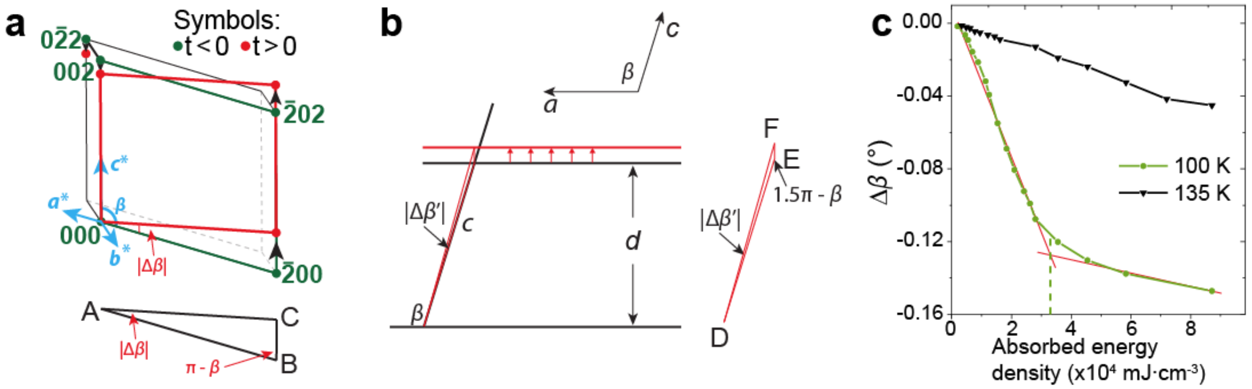

Supplementary Figure 5: Calculation of monoclinic angle change ($\Delta\beta$) and its dependence on absorbed energy density. **a**, Changes of β based on 002 and $\bar{2}02$ peak shift. **b**, β decrease due to an interlayer expansion (see Supplementary Note 3). **c**, Maximal $\Delta\beta$ at 100 K and 135 K as a function of absorbed energy density. The green dashed line indicates the threshold fluence at 100 K, as determined from the crossing of the linear trends (thin red lines) extrapolated from the low- and high-fluence limit.

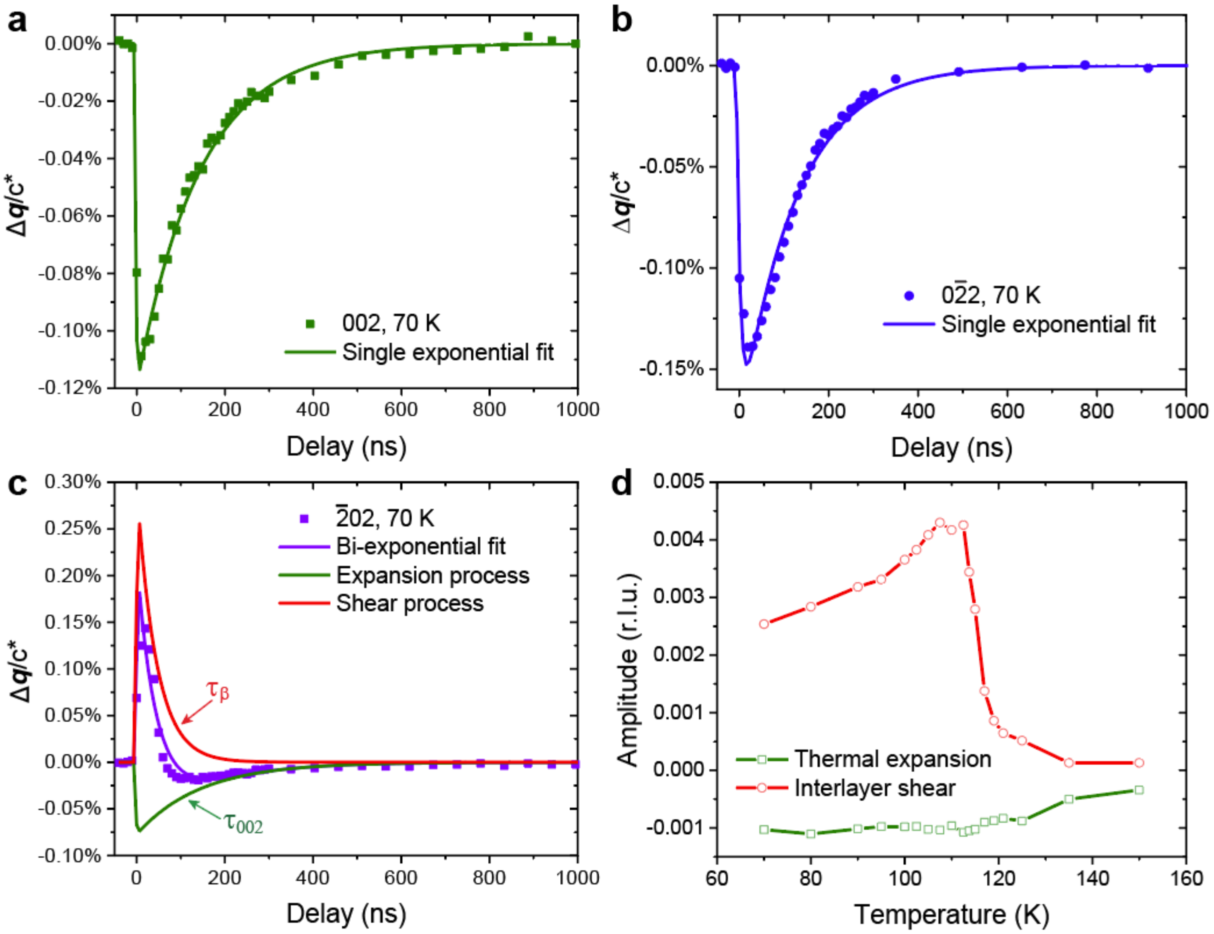

Supplementary Figure 6: Fitting of structural dynamics of $FePS_3$ by exponential decay functions. **a & b**, Single-exponential decay fitting of 002 and $0\bar{2}2$ peak at 70 K, respectively. **c**, Bi-exponential decay fitting of $\bar{2}02$ peak at 70 K. The green curve corresponds to the relaxation of interlayer expansion measured by the 002 Bragg peak while red curve corresponds to the relaxation of the β angle change. Purple curve is the sum of green and red curves. **d**, Comparison of the fitted amplitudes for thermal expansion (green curve) and interlayer shear (red curve) measured by 002 and $\bar{2}02$ peak as a function of the temperature.

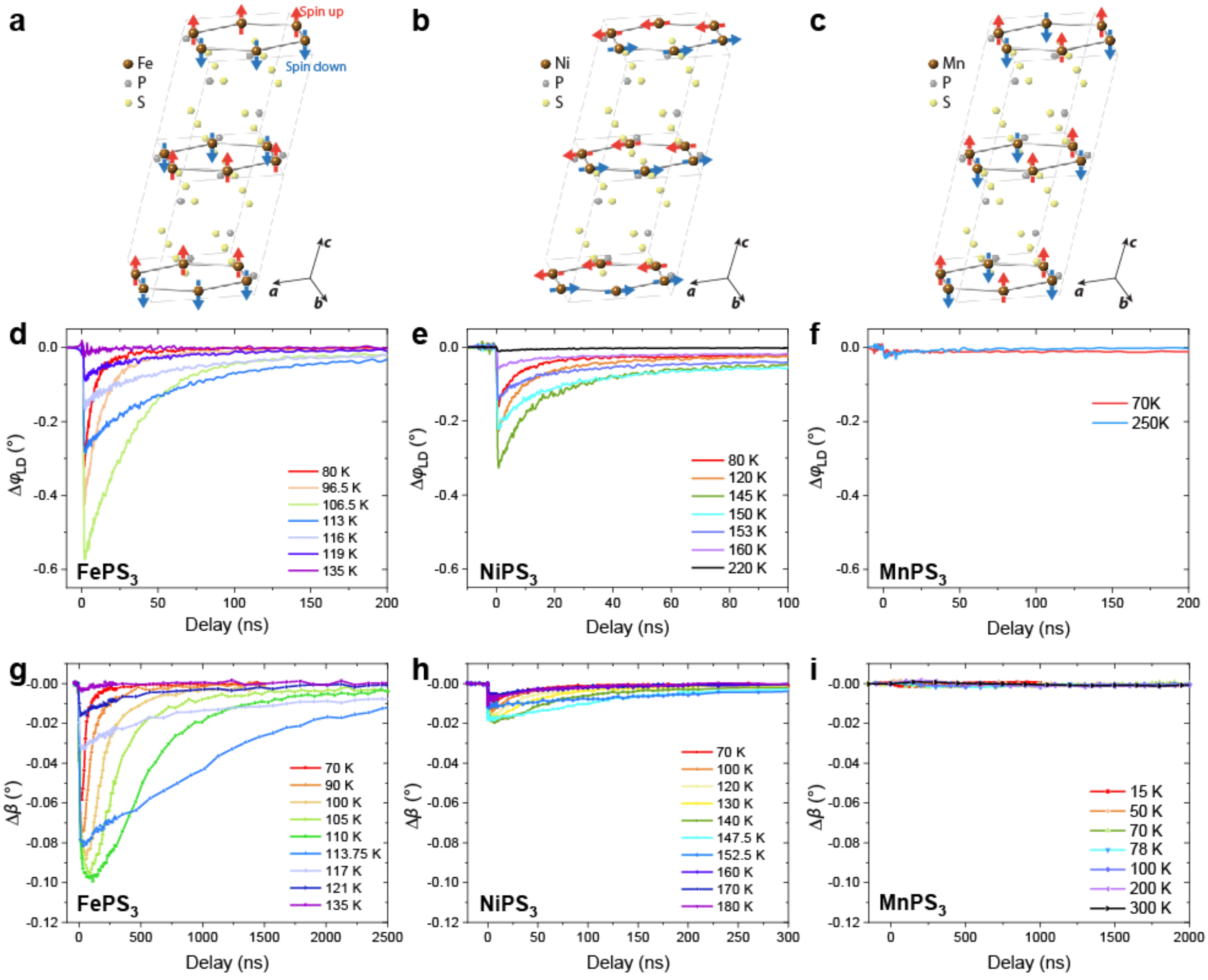

Supplementary Figure 7: Comparison of $\Delta\varphi_{LD}$ and $\Delta\beta$ dynamics in MPS3 (M = Fe, Ni, Mn). **a-c**, Crystal and magnetic structures of $FePS_3$, $NiPS_3$, and $MnPS_3$, respectively. **d-f**, $\Delta\varphi_{LD}$ dynamics in $FePS_3$, $NiPS_3$, and $MnPS_3$, respectively. **g-i**, $\Delta\beta$ dynamics in $FePS_3$, $NiPS_3$, and $MnPS_3$, respectively. The $\Delta\varphi_{LD}$ and $\Delta\beta$ dynamics are plotted in the same scale for the three compounds for comparison. The sample thicknesses are the following: $FePS_3$: 60 nm (**d**) and 1300 nm (**g**). $NiPS_3$: 40 nm (**e**) and 196 nm (**h**). $MnPS_3$: 50 nm (**f**) and 1100 nm (**i**). The sample thicknesses were chosen for optimizing the signal-to-noise ratios of the corresponding x-ray and optical probes. It is an extrinsic factor with a linear relation to the absolute delay time. But the thickness does not affect the scaling exponents as shown in Fig.3. The pump fluence for all the OLD measurements was ~1.0 mJ·cm^{-2} (400 nm, 1 kHz repetition rate). The pump fluences for the XRD measurements was set such that the interlayer thermal expansion was similar ($\Delta c^*/c^* \approx 0.05\%$) for all three compounds.

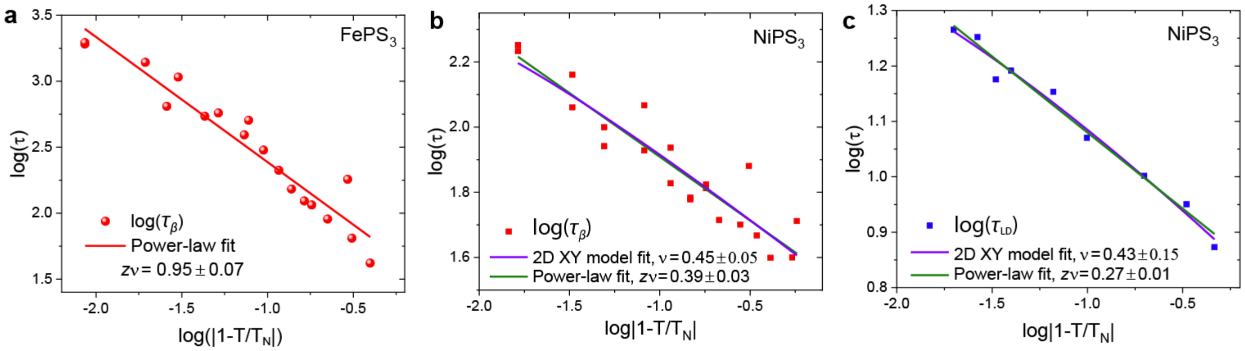

Supplementary Figure 8: Fitting of decay time as a function of temperature for $FePS_3$ and $NiPS_3$. **a**, Power-law fitting of τ_β for 1.3 μm-thick $FePS_3$. **b-c**, Power-law and 2D XY model fitting of τ_β (**b**) and τ_{LD} (**c**) for $NiPS_3$. Details of the fitting can be found in Supplementary Note 5. The quoted value of $z\nu$=0.33 for $NiPS_3$ in the main text is the average value shown in **b** and **c**. The quoted errors are fitting errors.

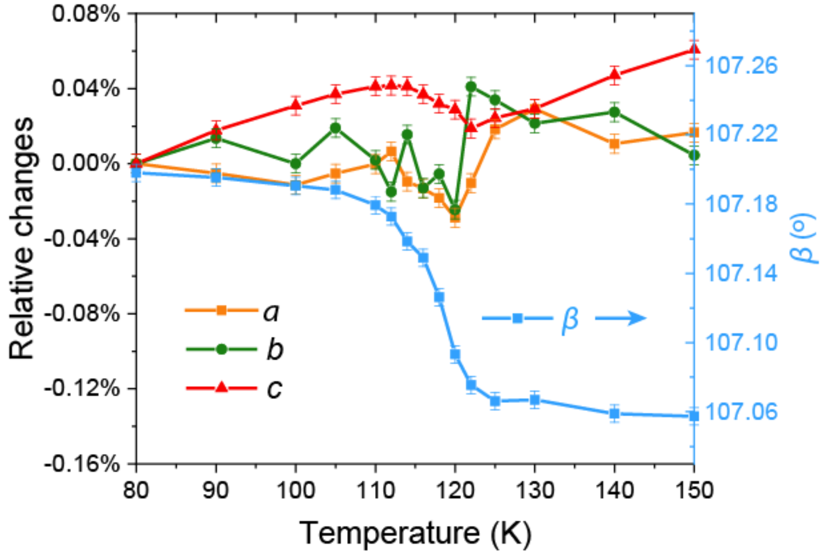

Supplementary Figure 9: Temperature dependence of the lattice parameters *a*, *b*, *c*, and β based on the single-crystal XRD of $FePS_3$. The error bars indicate the uncertainties in determining the HKL values of the refined Bragg peaks.

Supplementary Note 1: Comparison of peak center of mass shift and HKL scans

The time-resolved XRD measurements were performed in such a scheme that the sample and the detector were fixed while the diffraction peak was recorded on an area detector as a function of time delays. We show below that the change of the center of mass on the detector at the fixed incident angle in our experimental setup can be used to measure the Bragg peak shift in reciprocal space.

As shown in Supplementary Figure 2a, before time zero, the Bragg condition is satisfied, i.e., $\alpha = \beta = \theta_b$, where α, β, θ_b are the incident angle, exit angle, and Bragg angle, respectively. After laser excitation, the change of atomic plane spacing results in a change of the Bragg condition. As shown in Supplementary Figure 2b, the green and blue disks represent the finite-size Bragg peak. Δθ is the measured angular peak shift on the detector from point A to B. ∠AOC = $2\theta_b$ is the 2θ angle before laser excitation. ∠BOC is the 2θ angle after laser excitation. After laser excitation, ∠BO′C is the theoretical 2θ angle satisfying the Bragg condition, which can be calculated based on the peak shift on the detector. Based on trigonometry, one obtains

$$\angle \mathrm{BO'C} = 2 * \sin^{-1}\left(\frac{\mathrm{BC}}{2k}\right) = 2\sin^{-1}(\sin\theta_b - \frac{1}{2\sin\left(\frac{\pi}{2}+\theta_b-\Delta\theta\right)}\sin\Delta\theta\ )$$

In the experiment, θ_b for 002 peak is 10.15°. The measured peak shift on the detector at +5 ns corresponds to $|\Delta 2\theta|$ = 0.01°. The theoretical angular shift $|\Delta 2\theta|$ = $2\theta_b$ - ∠BO′C = 0.0103°, 3% larger than the measured peak shift of 0.01°. To confirm that the systematic error of measuring Bragg peak shift using diffraction peak center of mass change is negligible, the extracted shifts of 002 and $\bar{2}02$ peak were compared with the scans along the miller index L direction (L scan) using SPEC from Certified Scientific Software (Supplementary Figure 2c). As the figure shows, for both 002 and $\bar{2}02$ peaks, the peak center of mass shift is consistent with the L scan.

Supplementary Note 2: Estimation of transient temperature rise and simulation of thermal relaxation

The sample temperature rise upon laser excitation was estimated based on the interlayer lattice expansion. As shown in Supplementary Figure 4a, the measured change of c^* (interlayer spacing $d = \frac{2\pi}{c^*}$) follows a linear dependence on the temperature above and below T_N. The kink at T_N indicates a structural phase transition. The interlayer thermal expansion coefficient was estimated to be $1.818 \times 10^{-5}\ \mathrm{K}^{-1}$. In the pump-probe measurements, at a static temperature of 80 K, the measured Bragg peak shift $\Delta c^*/c^*$ = –0.055% corresponds to a transient temperature increase of 30 K, which corresponds to an incident fluence of 5.7 $\mathrm{mJ \cdot cm^{-2}}$ on the 1.3 μm-thick sample.

A one-dimensional (1D) thermal transport model was employed to simulate the cooling of the sample including the latent heat during the phase transition, following the method detailed in Ref.[2]. Using the following parameters: sample interlayer thermal conductivity[3] $k = 0.85\ \mathrm{W \cdot m^{-1} \cdot K^{-1}}$, sample-substrate interfacial thermal conductivity G=0.17 $\mathrm{W \cdot m^{-1} \cdot K^{-1}}$, the measured specific heat curve[1], the simulation results were summarized in Supplementary Figure 4b and captured the key features of experimental data. The thermal dissipation time gradually increases with sample temperature and peaks at 110 K, rather than T_N. The linear dependence of thermal relaxation time on sample thickness was also reproduced (Supplementary Figure 4c).

Supplementary Note 3: Calculation of monoclinic angle β change

$\Delta\beta$ was calculated based on the 002 and $\bar{2}02$ peak shift. As shown in Supplementary Figure 5a, the 002 peak shift to the **-c*** direction while the $\bar{2}02$ peak shift to +**c*** direction. The difference between the two peak shifts is directly related to β change. Based on the trigonometry shown in Supplementary Figure 5a, $|\Delta\beta| = \sin^{-1}(\frac{BC}{\sqrt{AB^2+BC^2-2*AB*BC*\cos(\pi-\beta)}}\sin\beta)$. In a small-angle approximation: $|\Delta\beta| \approx \frac{BC}{\sqrt{AB^2+BC^2+2*AB*BC*\cos\beta}}\sin\beta \propto BC$. The maximum BC we observed was 0.43%c^* at 110 K, corresponding to $|\Delta\beta|$ =0.10° decrease as shown in Fig. 2d and Fig. 3c. The laser-induced β decrease of 0.1° is consistent with the temperature-dependent XRD results, as shown in Supplementary Figure 9.

Since the crystal structure is monoclinic, an interlayer expansion with no interlayer shear can also lead to a decrease of β. Based on the trigonometry shown in Supplementary Figure 5b, $|\Delta\beta'| = \sin^{-1}(\frac{EF}{DF}\sin(3\pi/2-\beta))$. Using a small-angle approximation, $|\Delta\beta'| \approx \frac{EF}{DF}\sin(3\pi/2-\beta) \propto EF$. With EF = 0.05%$d$, we obtain $|\Delta\beta'|$ = 0.008°, suggesting that when the interlayer spacing increases by 0.05%, β decreases by 0.008° effectively, which is 12.5 times smaller than 0.1° β change across the phase transition.

The β change due to interlayer expansion was also observed experimentally. As shown in Supplementary Figure 5c, at the sample temperature of 135 K, $\Delta\beta$ increases linearly with the absorbed energy shown by the black curve. At the sample temperature of 100 K, the slope of β change as a function of absorbed energy (the green curve) is 10 times larger than that measured at 135 K, corresponding to the ~10 times difference between total β change and the interlayer expansion-induced β change. Above the threshold of the absorbed energy density 3.3×10^4 mJ $\cdot$ cm^{-3}, the slope of the green curve matches the black curve measured as 135 K, suggesting that there is no longer interlayer shear and the β change purely comes from interlayer expansion.

Supplementary Note 4: **Fitting of the time-dependent phenomena**

The relaxation time of XRD and OLD dynamics were extracted based on exponential function fitting. A single exponential decay function was used for fitting the time-dependent relaxation measured by 002 and $0\bar{2}2$ Bragg peak, as well as OLD, while a bi-exponential decay function was used to fit the dynamics measured by $\bar{2}02$ Bragg peak.

The single exponential decay function is: $\left(1-e^{-\frac{t}{\tau_r}}\right)Ae^{-\frac{t}{\tau_d}}H(t)$, where A is the amplitude, t is the time delay, τ_r and τ_d are the rise and decay time constants, respectively. $H(t)$ is a step function that equals 0 when $t < 0$ and 1 when $t > 0$. To reduce the fitting parameters, the rise time of the dynamics, represented by τ_r, was set to be a constant of 0.1 ns which matches the x-ray pulse duration. The amplitude A and decay time constant τ_d were the fitting parameters. A representative fitting result is shown in Supplementary Figure 6a & b.

The bi-exponential decay function for fitting the dynamics of $\bar{2}02$ peak is: $\left(1-e^{-\frac{t}{\tau_r}}\right)(A_1e^{-\frac{t}{\tau_{002}}}+A_2e^{-\frac{t}{\tau_\beta}})H(t)$. The two terms $A_1e^{-\frac{t}{\tau_{002}}}$ and $A_2e^{-\frac{t}{\tau_\beta}}$ describe the relaxation of the interlayer expansion and shear, respectively. The parameters of the first term were obtained from the fitting of the dynamics measured by 002 Bragg peak, while the amplitude and time constant of the second component were obtained by the least-squares fitting. At temperatures near

T_N, the rise time of $\bar{2}02$ peak is longer than 0.1 ns so the rise time τ_r was also a fitting parameter, which does not affect the fitting results of the relaxation time constant A representative fitting result is shown in Supplementary Figure 6c. Below T_N, the interlayer shear has a much larger amplitude than thermal expansion (Supplementary Figure 6d) and dominates the relaxation process.

Supplementary Note 5: Fitting of the critical exponents

The divergence in of both τ_β and τ_{LD} as a function of temperature were fitted by a power-law function: $\tau = A|1 - \frac{T}{T_N}|^{-z\nu}$, where *A* is the amplitude, *z* and *v* are the dynamical exponent and critical exponent of correlation length, respectively, T is the sample temperature. To better determine the sample temperature for each measurement, we first fit the data in a linear scale with three fitting parameters, A, T_N, and $z\nu$, which yield an initial value of T_N. We noted that this value has an offset of ±2 K with respect to T_N, which is attributed to the systematic error in the temperature measurements under laser illumination at different experimental conditions. Then, for better fitting of $z\nu$, the value of T_N was fixed in the fitting procedure using the equation in the log scale: $\log\tau = -z\nu\log\left|1 - \frac{T}{T_N}\right| + \log A$, with only two fitting parameters: $z\nu$ and A. The fitted $z\nu$ values were summarized in Supplementary Table 1 for samples with various thicknesses, with a representative fit shown in Figure 8a.

The width of the temperature range in which the slowing down is observed can be determined by the temperature at which the critical fluctuation is larger than the thermal fluctuation. This region spans from 105 K to 117 K (Fig. 2e) as the recovery of lattice becomes much longer than the thermal recovery. The width of this region is wider than those observed such as in ultrathin Fe films[4] due to weak vdW interaction and large spin fluctuations in vdW magnets.

**Measurement**	**Sample thickness (nm)**	**zv**
XRD, β	60	0.95 ± 0.12
XRD, β	200	1.15 ± 0.27
XRD, β	1300	0.95 ± 0.07
OLD, φ_{LD}	60	1.00 ± 0.04

Supplementary Table 1, Summary of critical exponents for different measurements in $FePS_3$. The error bar of $z\nu$ represents the standard deviation of the power-law fitting.

For $NiPS_3$, a 2D XXZ model system[5], the relaxation time as a function of temperature can take the following form[6]: $\tau = a|1 - \frac{T}{T_N}|^{-\nu} e^{bz|1-\frac{T}{T_N}|^{-\nu}}$, where *a*, *b* are constants, *z* is the dynamical exponent, *v* is the critical exponent of the correlation length. For 2D XY model fitting, only *v* rather than z*v* was obtained from the fitting (Supplementary Figure 8b & c). The fitted value was $\nu = 0.44 \pm 0.11$, close to the theoretical value of $\nu = 0.5$ (Ref.[6]).

As shown in Supplementary Figure 7, the shear amplitude and the magnetic order measured by OLD are larger in $FePS_3$ than that in $NiPS_3$. A possible explanation is the different electron configurations of the corresponding ions. For example, the Fe^{2+} ion has d^6 configuration,

which leaves the minority t_{2g} manifold partially filled, but the Ni^{2+} ion has d^8 configuration, so the minority t_{2g} manifold is fully filled, which quenches the angular momentum and in general leads to weaker spin-lattice coupling compared to partially filled d-orbitals. In addition, the strength of critical slowing down is also different: the recovery time of the shear motion in $FePS_3$ increased by two orders of magnitude and only increased by one order of magnitude in $NiPS_3$. This is likely due to the different spin configurations in these two compounds. The zigzag spin order is Ising type and 2D XY type in $FePS_3$ and $NiPS_3$ respectively, which have distinct scaling exponents and universality classes.

Supplementary Note 6: Temperature-dependent single-crystal XRD for $FePS_3$

We performed single-crystal XRD as a function of temperature at the 7ID-C beamline of the Advanced Photon Source. During the measurement, the orientation matrix was optimized and fixed at 80 K. The H, K, L values of multiple Bragg peaks (002, 004, $\bar{2}04$, $\bar{4}24$) were recorded at each temperature and used to calculate the a, b, c, and β, as summarized in Supplementary Figure 9.

We note that previous reports of the structural transition in $FePS_3$ used the powder XRD method[7,8] did not report monoclinic angle β change as a function of temperature. To cross check our single-crystal structure refinement results, we also performed high-resolution powder XRD at 11BM beamline at the Advanced Photon Source. The powder XRD results yield a distinct β change of 0.02° across T_N. This is a factor of 5 smaller than the single-crystal result. We ascribe this discrepancy to the sample preparation for powder diffraction, in which the samples were grinded into powder and attached to the Kapton tubes with vacuum grease. This process likely introduced strains in the sample, resulting in a smaller β change in powder XRD data.

Supplementary References

1. Takano, Y. *et al.* Magnetic properties and specific heat of MPS_3 (M=Mn, Fe, Zn). *J. Magn. Magn. Mater.* **272–276**, E593–E595 (2004).
2. Wen, H. *et al.* Structural and electronic recovery pathways of a photoexcited ultrathin VO_2 film. *Phys. Rev. B* **88**, 165424 (2013).
3. Kargar, F. *et al.* Phonon and thermal properties of quasi-two-dimensional $FePS_3$ and $MnPS_3$ antiferromagnetic semiconductors. *ACS Nano* **14**, 2424–2435 (2020).
4. Dunlavy, M. J. & Venus, D. Critical slowing down in the two-dimensional Ising model measured using ferromagnetic ultrathin films. *Phys. Rev. B* **71**, 144406 (2005).
5. Kim, K. *et al.* Suppression of magnetic ordering in XXZ-type antiferromagnetic monolayer $NiPS_3$. *Nat. Commun.* **10**, 345 (2019).
6. Jelić, A. & Cugliandolo, L. F. Quench dynamics of the 2d XY model. *J. Stat. Mech.* **2011**, P02032 (2011).
7. Jernberg, P., Bjarman, S., Wappling R. $FePS_3$: a first-order phase transition in a '2D' Ising antiferromagnet. *J. Magn. Magn. Mater.* **46**, 178-190 (1984).
8. Murayama, C. *et al.* Crystallographic features related to a van der Waals coupling in the layered chalcogenide $FePS_3$. *J. Appl. Phys.* **120**, 142114 (2016).
